# Du Traitement Moral de la Folie

**Published:** 1841-04-01

**Authors:** 


### Du Traitement Moral de la Folie. Par M. *Leuret*. 8vo. pp. 462. 1840.

The scope of the present work, from the pen of one of the physicians of the Bicetre Hospice at Paris, is to endeavour to correct many of what he deems to be prevailing errors in the management of certain forms of mental alienation. Its sum and substance are comprised in the following three propositions, which are laid down in one of the opening pages, and which are subsequently illustrated at great length by reference to the recorded opinions of other authors, and to the extensive experience of M. *Leuret* himself.

1. If it be true that insanity always depends upon, or is connected with, some lesion of the encephalon, it must surely be admitted that as yet we are completely ignorant either of the nature, or of the exact seat, of the lesion.

2. The moral treatment of insanity, as recommended and practised by the best writers on the subject, has been viewed by them only as an auxiliary or adjunct to the more important remedial means, the physical treatment.

3. In my opinion, on the contrary, insanity, when it is not associated with corporeal disease or suffering, is most efficaciously relieved, or even cured, by appropriate moral treatment; whereas physical means, under such circumstances, are of little or of no avail.

M. *Leuret* proceeds to adduce numerous arguments and illustrations in proof of these three positions; premising, however, this important caution to his readers, that these positions are meant to apply only to insanity, or disturbance of the intellectual and moral faculties, when it is uncomplicated, or, in other words, unattended with symptoms of corporeal disease. "For," says he, "if there be present at the same time paralysis, apathy, agitation, loquacity, fever, &c. we have reason to infer that there is a physical lesion somewhere, and we must therefore have recourse to physical means to relieve it; but in simple derangements of the reason or passions, on the contrary, in cases where the insanity exists alone and without complication, it is moral treatment that is most required."

Let our readers therefore bear in mind this precautionary remark, while we submit to their attention the prominent contents of our author's work.

To prove his *first position*—that, if insanity depends upon any organic lesion of the encephalon, we are as yet completely ignorant as to wherein this lesion consists—he takes a review of the numerous morbid changes which have been described by various writers on the subject, and shews how utterly discordant are their opinions and assertions upon the subject in question.

For example, he shews from the writings of *Greding*, *Haslam*, and *Bertolini*, that there is no uniform change in the thickness of the cranial bones as has been alleged by some authors;—from those of the writers now named, as well as of *Bayle* and *Calmeil*, compared with the published researches of MM. *Louis* and *Chomel*, that, although lesions of the meninges of the brain are frequently found on the dissection of insane patients, these very lesions are not only seldom met with in *uncomplicated* and monomaniacal insanity, but are also frequently discovered in the bodies of persons who have never, at any period of life, exhibited any traces of mental derangement;—that sanguineous injection or hyperæmia of the cerebral substance cannot be regarded as a characteristic morbid lesion accompanying insanity; since, on the one hand, it is not uniformly present, and, on the other hand, it is very frequently observed after various chronic diseases of the body*—that hypertrophy and atrophy of the brain are only occasional,

* M. *Leuret* is far from denying that hyperæmia of the brain exerts a real the former more rarely than the latter, necroscopic phenomena—that œdema of the brain is a still more unfrequent phenomenon—that there is the greatest discordancy among authors as to the frequency of any increase or of decrease of density in the substance of the brain in cases of insanity, uncomplicated with manifest corporeal disease*—and, indeed, that the same assertion holds good of every other morbid alteration which has been described by authors.

He next alludes to the important fact that every writer on the subject, without exception, admits that in some cases of insanity no traces whatever of any alteration in the brain or its appendages are discoverable on the minutest examination.

Thus M. *Calmeil*, after mentioning that, in eight out of seventy-five cases of insanity, the encephalon was found on dissection to be altogether healthy, adds these words:...... "the anomalies of structure observed in the bodies of the insane are not in themselves sufficient to account for the state of alienation, because we sometimes meet with the same appearances in those who have never shewn any symptom of it."

M. *Esquirol*, too, most distinctly admits that dissection has quite failed in making known to him what is the physical cause of insanity; and M. *Lelut*, who of late years has prosecuted his pathological researches with the greatest assiduity, expresses the same opinion. M. *Heinroth*, the German translator of *Esquirol*'s works, even maintains that the brain is a stranger to the production of insanity!

But M. *Heinroth* is one of those spiritualist gentlemen who subtilise many ordinary physical phenomena, until we can scarcely recognise their existence: his opinion, therefore, is not likely to have much weight with the English reader.

M. *Leuret* makes a few remarks on the attempts made by *Gall*, and other phrenologists, to localise the different forms of insanity in different parts of the encephalon; but, in spite of the arguments and reasonings of MM. *Ferrus* and *Parchappe* among his own countrymen, and of Drs. *Ellis* and *Elliotson* in this country, he regards them as quite unsatisfactory, either for sound theory or judicious practice.

He sums up his remarks on the pathology of insanity with the following observations:—1, that physicians have, without any spirit of discrimination, accumulated or huddled together all the morbid changes which they have found, or believed to have found, in the brains of persons who have died insane; 2, that they have been in the habit of much too hastily attributing the disturbance of the intellectual and moral faculties to these real or sup-

---

influence in producing many of the pathological phenomena which are observed in insane patients. All that he contends for is, that it is not to this morbid condition that the mental or *psychical* symptoms can be justly attributable; and, consequently, that it is by no means a uniform necroscopic appearance in cases of simple uncomplicated insanity when the physical or corporeal health remains unaffected.

* Thus M. *Ferrus* has asserted that, in melancholy with a tendency to commit suicide, the brain is generally *excessively soft* and exsanguine, while M. *Cazauvieilh*, in his recent work Du Suicide, &c. 1838 states, that in seventeen dissections of suicides the substance of the brain was unusually firm.

posed changes of structure; 3, that they have too much neglected to take account of the changes which are compatible with the integrity of the mental faculties; and 4, that, as regards the changes said to be peculiar to the insane, the distinctions between the symptoms which are of a physical and those of a psychical or mental nature, during the life of the patient, has not been duly attended to.

"I do not wish it to be concluded," says he, "from these observations, that in my opinion, the brain does not experience any alteration in the insane, even in cases where the mental alienation appears to be free from any other morbid complication. I admit, in the production of insanity, the influence of certain physical causes; and I also admit that organic lesions of the brain are more frequent in the insane than in any other sort of patients. But then as to the nature of the alteration, which is the immediate cause of insanity, I assert that hitherto no one has been able to point it out. If it really exist, it must be similar to that which gives rise to dreams, which suggests the false convictions in persons of otherwise sound minds, and which excites the instincts and passions. On no occasion does it reveal itself by physical characters, and its nature is completely unknown."

The *second* chapter of the work is occupied with a summary review of the published opinions of the leading French writers on insanity during the last forty years on the important question, how far moral treatment avails to the mitigation or removal of the mental disorder. M. *Leuret* shews that by all, without exception, it has been regarded only as an auxiliary, or aid to the more important means of physical or corporeal medication.* In his opinion, the rule should be reversed; the moral should take the precedence of the physical treatment, in all cases where the insanity is not associated with obvious disturbance of the bodily health.

---

* Our author alludes to several recently published English works, which, like those of his own countrymen, are all, he says, too much occupied with instructions as to the mere physical treatment of the insane. He illustrates this by commenting on an instance of theomania, recorded by Sir Alex. Morrison, in his Cases of Mental Disease 1828. "The patient, whose health was good, thought that he was frequently conversing with spirits; to prevent him seeing them, he was directed to take pills of calomel and jalap. He then thought that he was God; the pills, and baths also, which had been administered, were discontinued. He became violent; forthwith he was shut up in his cell and cupped. He broke the windows; a dose of ipecacuan was given him. No change in his condition; camphor and hyosciamus were ordered. Such is a specimen of the unmeaning treatment pursued in a great number of lunatic establishments."

M. *Leuret*, however, acknowledges that one or two of the English writers have counselled more wisely. He alludes to Mr. *Tuke* (a description of the Retreat near York for insane persons, 1815) in the following commendatory terms: "He justly condemns an old practice at Bethlehem of bleeding and purging all the patients at regular periods; and endeavours to point out the inefficacy of physical remedies, while he strongly urges the importance of appropriate moral treatment. He wishes that the insane person be led to exercise himself a controul upon his own actions, and that his medical attendant endeavour to inspire him with various emotions, and occupy his mind with various ideas. In all this, I entirely agree with him; the only thing that I find fault with him is, that he has not distinguished, in reference to the treatment, the cases of simple uncomplicated insanity from those in which there is co-existent some lesion of the movements and of the sensibility."

This is the great aim and object of M. *Leuret's* work ; and certain it is that he has worked out his problem with very considerable ingenuity and success.

In a vast number of cases of monomaniacal or partial insanity, there is unquestionably not a little share of wilful obstinacy and petulance of temper blended with the existing mental delusion ; indeed, the patient himself will not unfrequently admit the folly of his vagaries when under the influence of strong hope or fear. This admission indeed may be for the moment only, when these feelings are worked upon ; and, whenever their influence ceases, all the former extravagances will re-possess the mind. But if, as M. *Leuret* remarks, we once succeed in frequently renewing the effects of the emotions thus excited, so that the patient himself begins to associate the idea of the indulgence of a favour, or of the infliction of a penalty, with certain phantasies of his mind, we shall find, in not a few instances, that these phantasies will soon have a fainter and fainter hold upon the attention, and will ultimately entirely vanish. It is by working on the hopes and fears of the patient—provided always the bodily health be perfectly good at the time—and by blending kindness with an authoritative firmness in all his demeanour, that the physician may best hope to acquire that influence over his patient's mind, which so often conduces to the restoration of its perfect sanity. Our author very justly remarks, that there are many points of analogy between the character of a monomaniac and of a froward and spoiled child. No one would think of physicking the latter for petulance of temper ; and it is no less absurd to endeavour to dispel the illusions of the former with purges and mercurials.

The means, to which M. *Leuret* chiefly trusts for acting on the fears of the insane, is the use of the cold douche bath. The force and size of the stream of water, the height from which it comes, the length of time it is to be continued, &c. must be varied according to circumstances. Most patients complain most bitterly of its use, and will make almost any concessions to escape its repetition ; but some remain quite indifferent about it. The aim of the physician should be so to manage its administration that the patient, while under the douche, will make an earnest promise to perform something that is required, or abstain from something that is forbidden.

"When I have once gained a concession," says M. *Leuret,* "I am not satisfied; I require new ones each day; the more that are granted, the more I require; and, if I see the hopes of a cure, I stop in my demands, only when this has been effected."

It is quite unnecessary to allude to the reproaches which have been thrown upon our author by many of his confreres for his alleged want of proper feeling towards his patients, and his endeavours to work so much upon their fears. If his cruelty does not go beyond giving them cold douche baths against their will, and to their very obvious annoyance, the most sentimental moralist will doubtless excuse him.

While insisting much upon the value of this mode of overawing many insane patients, M. *Leuret* is far from trusting to it alone in the management of various forms of mental alienation. He recommends that every means should be employed to divert and amuse their minds, and to withdraw their thoughts from their delusions by keeping both their mental and their bodily powers engaged. The value of out-door exercise, of walking, riding, or driving, of working in the garden, of engaging in various sports, and also of in-door amusements, such as music, billiards, the acting of plays, &c. is much dwelt upon. It is unnecessary to say more; as every one is now aware of the hurtful effects of long seclusion and silence even to people of sound minds:—what then must be their influence on the insane? M. *Leuret* recommends that the invalids should dine together. It is always well to keep up the rules strict of etiquette during every repast:—the attention is thus to a certain degree maintained, and, by the performance of the little courtesies to each other, the patients are accustomed to exercise a certain degree of controul over their own feelings. In large establishments, it is often useful, as well as very convenient, to divide the patients into several *messes*—giving to one the superintendance and preparation of the food for his division, after the manner practised on ship-board.

Having thus briefly stated the leading contents of M. *Leuret's* work, we shall extract a few of the cases, abridged, which he records in illustration of the sort of moral treatment recommended and adopted by him in public and in private practice.

CASE.—*Imprisonment for a political offence—production of the thought, accompanied with an hallucination of hearing* (?) ; *refusal to speak or to take food—cure by moral treatment.*

A man, 30 years of age, was admitted into the Bicetre in May 1838. When M. *Leuret* visited the hospital on the following morning, he found his patient pale, lying on his back, and most doggedly taciturn ; he would not answer a single question, and he had refused to taste either food or drink, not only since his admission, but for upwards of a week before. His pulse was natural, and nothing indicated any corporeal disorder.

As hitherto he had refused to answer any of the attendants, M. *Leuret* did nor address any question to him ; but, seeming to be quite indifferent whether he spoke or not, said in an authoritative tone—"This man must be made to drink:" and, immediately closing the patient's nostrils with one hand, he forced into his mouth a cupful of gruel with the other. In spite of some resistance, the gruel went down ; and M. *Leuret* enquired of the attendants if the *douche* was ready, as it must be used at once, if he spat out any thing that was given him. Already M. *Leuret* had acquired some influence over him, although he had not addressed a word to him.

In the course of the day, he passed urine in bed ; but, without reproaching him for this, he was merely ordered to swallow a cupful of broth ;—he was made to do so by using the same means as before. At the same time he was ordered to rise from bed ; as he did not move, the attendants at once drew him out, and put on his clothes: when dressed, he consented to stand up. He was led out into the garden, where a number of the patients, ranged in a chain, were engaged in passing stones, the one to the other. Poor *Urbain*, weak as he was, was placed in the middle of the chain, and when his neighbour held out a stone to him to be passed to the next on the other hand, he looked at it, smiled, and after a moment of hesitation, took it and passed it along. This was repeated several times, and before long he engaged in the work as cleverly as any of the others. While this was going on, a tureenful of soup was brought, and a spoon was given to each man. *Urbain* was invited by one of his neighbours to come and eat; he allowed himself to be led to the table, took a spoon, and began to eat as well as the rest. In the evening he refused to eat, and, instead of drinking what was offered to him, he took hold of the spitoon and swallowed all its contents.

Next day, he seemed somewhat better; but, strange to say, he was ordered by the visiting physician in attendance on this day to be cupped on the neck, have an aperient enema, and a tepid bath! and many kind exhortations were addressed to him. M. *Leuret* again took charge of him in a few days, and, instead of doctoring him with physic, bade him get out of bed, and carry some pitchers of water for the use of the ward: when he had done this work, a couple of boiled eggs and some bread were put into his pocket, and a cupful of milk was put at the side of his bed. When left alone he eat the eggs, and drank the milk.

Next day, he refused either to speak, or to take his food: the douche was therefore resorted to; he bore it at first without winking; but soon began to be distressed with it, and exclaimed for the first time, *Mein Gott! mein Gott!*

Without taking any notice of what he said, he was merely asked whether he would take food; he consented and swallowed what was put before him. For the next nine days, it required compulsion to make him take food. What was curious, and shewed how much real obstinacy is present in many such cases, was that he made no resistance to the introduction of an œsophageal tube into the stomach, but that, if a spoonful of food was put into his mouth, he at once spat it out.

On the ninth day, the obstinacy of *Urbain* being overcome, he consented both to speak and to eat his food. When asked what had induced him to refuse doing so before, he made no reply—probably from being aware of having no good reason to allege.

By degrees he became more and more submissive; and, in order to prevent him brooding over his own thoughts, he was kept almost constantly occupied in some way or another.

Two months after his admission into the hospice, he was permitted to leave.

The cause of the malady in this case was purely moral, having been induced by imprisonment in Germany for some political offence, and increased by subsequent distress and privations, when he made his escape into France. Such a case is surely not to be treated by cupping, blistering, and physic-taking, as is too often tried. It is by acting on the mind with firmness, and yet with kindness, that we can hope to overcome the gloom and obstinacy of such a patient.

M. *Leuret* mentions the case of a monomaniac in the Bicetre, who, in consequence of the utter inefficacy of all the means that had been tried, had been placed in the section of the incurables, and who had formed the resolution to perish from hunger. For three entire days he did not swallow a mouthful of anything. He was ordered to have the douche. When this was over, he spoke to M. *Leuret*, and asked him why he was treated so, adding that it was better to die of want, than live miserably in a hospital. "It rests with yourself not to live in a hospital; cease to act unreasonably; if you give over your sullenness, and begin to work and eat, you shall have your freedom."

"My freedom! when will you give me it?"

"In a month, if you choose."—"In a month? then I will eat." From this time his obstinacy ceased; and at the end of the month, the promise, which had been given in the bath-house, was faithfully fulfilled.

M. *Leuret* alludes to another case, that of a young lady who had formed a similar resolution to kill herself by fasting. Food however was introduced into the stomach by means of the œsophageal tube: but, if any was put into her mouth, she at once rejected it. No decided progress was made in overcoming her insane intention, until a little *ruse* was practised. Her family were requested to go to M. *Esquirol's* establishment, where she had been living, and, without making any allusion to either her past or present condition, to invite her to accompany them to Versailles. She went with them; and, after walking about in the gardens for some time, the whole party repaired to a restaurateur's; she sat down like the rest; hesitated for a moment; but, no notice being taken of her, she began to eat: from that time she never refused to take her food, all her gloomy ideas vanished, and she returned home, enjoying her perfect reason.

"Here," says M. *Leuret*, "the practice followed was quite independent of pathological anatomy; and indeed it is the only rational one in all cases of simple, uncomplicated insanity."

Case.—*Disappointed love—hallucinations of sight and hearing; ambitious ideas—temporising useless; moral treatment successful—duration of the disease, nearly four months.*

A man, 31 years of age, was admitted into the Bicetre on the 17th Sept. 1839. His tale was, that for two months he had been desperately enamoured with a fair seamstress, who at first treated him scornfully, but afterwards became as loving as possible. The poor fellow's head was upset by his good luck; and, from one absurdity to another, he at length worked himself into the assurance that his "ladye love" was not one of earthly mould, but a heavenly messenger, who visited him in his dreams. However, as she would never consent to marry him, he fell into a state of the gloomiest melancholy, and committed the greatest absurdities, until at length he was brought to the Bicetre. His bodily health was quite good, and being aware where he was, he most urgently implored to be discharged, as, he said, he had to attend to his business. At length, he became reconciled, and was very calm; he eat and slept well; but he would not work, alleging at one time that a prophet never works, and at another that he was soon to be liberated, and therefore that there was no occasion to begin any work. He was amused with the tales and conduct of the other inmates, and seemed to be quite aware of their absurdities; but as for himself, "he knows what he knows; fools act extravagantly, but no one can say that of him. He has never spoken a word that is not altogether true, and, although he is not believed, yet it will be found out sooner or later that he has been in the right; he will never draw back from what he has said, nor will he consent to work like a drudge for any one."

For two months no change took place in his delusions; but, as he still refused to work, M. *Leuret* thought that it was high time that a decided attempt should be made to compel him to do so. He was therefore ordered to have the douche. From this time, he offered to work at any carpentry, but positively refused to do anything else.

"I have no carpentry work to give you; you must labour in the garden, like your companions. You shall have a spade, and let me see you set about using it immediately."

The fear of another douche made him submit. While engaged in working, M. *Leuret* spoke to him about his sweetheart, her visits to him as an angel at night, &c.; and he now confessed that it was all a delusion, that he no longer believed in what he formerly said, and that he was determined no more to think about what he now knew to be mere folly. He kept his word, and was in consequence dismissed in about a month afterwards.

"Some may object," says M. *Leuret*, "to my conclusions, and assert that a promise extorted from an insane person by fear is not likely to have any influence over him, when he is left to himself. But this is really not the case, provided the fulfilment of the promise has been kept up for a considerable time, before the restraint is removed. The renunciation of the foolish ideas is at first, I admit, merely on the lips; but when the person finds that along with such a renunciation he must act consistently, the mind is gradually withdrawn from its errors, and, if kept occupied, it will often revert into the channel of sounder thought. I have known many persons, who, long after their cure, when they called to mind their hallucinations, rejected them with all their energy, from the circumstance of associating with their existence the treatment which had been followed......... The conclusion from what we have now stated is, that in numerous cases we may succeed, by means of simple moral treatment, in dispelling the illusions of the insane."

CASE.—*Sedentary and Inactive Life; heating diet—fear of damnation; perverted sensations—Cure effected by the use of cold baths, exercise, &c.—Duration of the illness, eight months,*

A young married lady, who had been the ornament of the society in the capital, retired with her husband into a sequestered part of the country, where, there being little to occupy her mind or feelings, she passed much of her time in bed and inactivity. After a nervous attack, her mind seemed to be much shaken; she was haunted with the fears of religion and believed that she was eternally lost. She was put on a mild unirritating diet and gradually recovered. Soon afterwards, however, she was seized with violent palpitations of the heart, for which leeches were applied on the precordial region. She became more and more restless, and all her former fears returned with double force. Although she had received absolution from her confessor, she did not believe that her sins had been forgiven. Some days she sat in a corner of her room and would not speak to any one, and at other times she ran about the fields screaming in the wildest manner. She was taken to Paris and boarded in a convent of the *religieuses hospitalieres.* Here every means of spiritual consolation—as private and public prayers, pious songs, visits to various chapels, counting of rosaries, kissing of holy relics, absolutions (what mummery !), &c. were lavished upon her; but without avail. She was, therefore, transferred to a *maison de santé*, where she was visited by M. *Leuret*. After relating her complaints she told him that no one could cure her, for that she was inevitably doomed to hell, and that all that she wished was to be left alone in a silent apartment, where others might not suffer from witnessing her agonies. Her bodily health seemed to be quite good; the catamenia were regular: she was forty-two years of age, and had been six months ill. M. *Leuret* assured her that she would get better, and, instead of leaving her in a room by herself, gave orders that she should be placed in a part of the building where there were upwards of a dozen persons. He told her that he would change her quarters in the course of a few days, if he deemed it proper, and at the same time he impressed upon her that, if she uttered any screams during the night, it would be necessary for her health that she should be taken to the bath: "I count much," said he, "on the cold bath long continued to calm your nervous excitement." At night she began to scream and shout aloud, blaming M. *Leuret* at the same time that he had left her in a room where her cries disturbed other patients. She was, therefore, at once taken to the cold bath, in which she was kept much against her will for some time: she gradually became quiet, was taken back to bed, and then fell asleep. Next night she was again noisy, and the cold bath was repeated with the same effects as before. On the following noon, a third bath was given in consequence of her noisy restlessness.—"But, Sir, to-night again; I have already been in the bath for four hours." "Well, madam, other four hours are necessary; it is by the violence of the disorder that the frequency and the length of the baths are determined." From this time Mad. E. understood that the only way to escape the baths was to give over screaming; and the effects were soon apparent, for in the course of a few days she had ceased entirely from making any noise. Although now much less gloomy and unhappy than she had hitherto been, the cause of her crying still continued; she said "Every morning, soon after waking, I feel a most oppressive weight on all my limbs, while this part (pointing to her heart) feels quite empty: all my distress is owing to ten devils who come to lay hold of me. My moral heart is gone; I love nothing now; for the condemned do not know what it is to love. The chain which knit my heart to heaven is broken; my prayers cannot reach God; I am lost, I am lost." "Have you ever seen or heard any thing *en dehors de* your ordinary sensations?"—"Yes, once I heard a voice which said—thou art lost." "When did you hear this voice?"—"A long time ago, at the beginning of my illness." "Whence did it come?"—"From the inside of my body." "How were you certain that it was really a voice, and not merely an idea?"—"Eh, Mon Dieu, by the noise?" "Was it a noise produced at the same time as the idea, or was it a sound of the voice?"—"A sound of the voice: I do not know how the nurse, who was with me, did not hear it." "These devils whom you hear, have you never seen them?"—"No."

Madame E. continued still gloomy and seemingly most wretched. The only plan to dispossess her mind of her delusions was to keep her occupied, and an appeal was, therefore, made to her kind feelings to engage her in preparing some lint for a poor man who had met with an accident. Her fingers at first would scarcely move; but, when she saw others doing it, she at once set to and worked most diligently—for she was naturally of a benevolent and charitable disposition. The first step was thus made; she let us see that she could work, and she was convinced of it herself; the object now was to find a motive to induce her to do so every day. The fear of the cold bath furnished this motive; for M. *Leuret*, finding her one day gloomy and silent, began to scold the nurse for not giving her the cold bath every day, adding that the use of it was absolutely necessary for every one that did not work at something. This had completely the desired effect; she worked a great deal with her needle; her mind was thus diverted; gradually she entered into conversation, resumed her music, and actually began to laugh at her past delusions. Within two months Madame E. was able to return home; she travelled about for some time; and ever since, a period of seven years, she has had no return of her mental malady.

In commenting on the preceding case, M. *Leuret* impresses on his readers the importance of a physician accommodating his manners and mode of treatment to the peculiarities of each case. "He must strive to make himself master of all his patients; but this he cannot do unless he varies and multiplies his means of action in innumerable ways. According as need be, he should employ either a firm and even a rude or a conciliating manner, either condescendence or despotism; he must flatter in one instance, and check in another, certain passions; now lay a little stratagem and now act with the utmost candour and seeming confidence; in one word, seek in the minds and tempers of those he wishes to cure for some spring or lever, which, once set in motion, may restore to the mental faculties the energy or the rectitude which they have lost."

Case.—*Vanity and Love—demand of a princess in marriage—Mental Alienation—carried almost to Mania—Moral Treatment; Cure—duration of the illness, eight months.*

A man, 37 years of age, and who, although the son of an old general of Napoleon, had been for a length of time in a draper's shop, where he had uniformly shewn great attention to business, became all at once smitten with desperate love for one of the daughters of Louis Phillippe. He followed her carriage wherever she went, tried to attract her notice by dressing in the most fashionable style, and even sent presents of gloves, &c. to her. He became at length so extravagant as to stand at one of the corners of the palace and kept moving his hand from his lips to and fro, as if wafting kisses to the fair princess.

When taken to the Bicetre, he was guarded in his answers: but, whenever any allusion was made to the royal family, he indicated by his gestures that his ideas were constantly recurring to that subject.

M. *Leuret* did not take any notice of him for some time, except every now and then expressing his surprise that he should have given up the respectable occupation of a merchant to become a dependent upon charity. But as he continued in the same state for several weeks after his admission, he was ordered to have the cold bath, and also to work every day in the garden. He refused; a douche was ordered; and immediately afterwards M. *Leuret* remonstrated with him on the absurdity of his conduct.

This time he began to listen, and requested liberty to write the history of his case. In the report, after alluding to his devoted attachment to the royal family, and his long and ineffectual attempts to obtain a situation in the palace, he expressed his regret that he had ever written to the princess, or annoyed her in any way; attributed his delusions and the circumstance of his being confined in the Bicetre to anxiety of mind, and exposure to the hot sun on one day that he walked to *Eu*, to solicit an interview with Baron Athalin; and requested to be permitted to return to his commercial speculations.

M. *Leuret* was satisfied that a very considerable amendment had already taken place, but insisted that he should remain some time longer, and his attention be kept occupied with various employments. At length seven weeks after his admission, he was discharged as cured. M. *Leuret* saw him five months afterwards, and could observe no traces of insanity in his conversation or in his conduct.

CASE.—*Habits of Intoxication: ambitious ideas; project to reform the society of the world—moral treatment; cure—duration of the illness, several months.*

A man, 37 years of age, lost his situation as clerk in a public office in the country, in consequence of frequent inebriety, although he always professed the most rigid religious principles.

He came to Paris, and became a joint editor of some publication: his irregular habits still continuing, he again became embarrassed, and partially lost his employment a second time. All the while, however, he continued to be most strict in his religious observances, and in his sober moments condemned his conduct as most improper. He became more and more inconsistent in his actions, and at length discovered that he was a man of extraordinary genius who was destined to revolutionise the world. He was taken to the Bicetre, where he was kept, as a matter of course, quiet, sober, and away from his nonsensical companions. As no change, however, took place in his extravagant notions, he was ordered to have the douche. While in the bath, M. *Leuret* related to the assistants how inconsistent he was; for, while he professed to be very religious, he was a drunkard, a liar, and a conceited puppy. The effect of the douche was instantaneous; he immediately renounced all his ideas of regenerating the world. To try how far his promises were sincere, M. *Leuret* asked him whether a second douche might not be of use in confirming his good resolutions; but, as he gave the most positive assurances that he would never relapse into his former errors, and combated these errors with the most rational arguments, the bath was not repeated. He kept his word; remained for a month in the hospital, and was then discharged.

M. *Leuret* saw him several times afterwards; and, although his head was certainly not very strong, he did not exhibit any symptoms of mental derangement.

We might have selected other cases, related by M. *Leuret*, in which the insanity had been of longer duration, and was more confirmed than in any that we have given. But these will suffice to illustrate his method of treating some of its numerous forms, and to show how much may be done by the firm adoption of a judicious moral regimen in cases, where the mental disturbance is not accompanied with any bodily disorder.

